# Treatment strategy for compartment syndrome at multiple regions due to injuries caused by a tree fall: a case report

**DOI:** 10.1186/s12245-024-00675-5

**Published:** 2024-07-15

**Authors:** Tomotaka Miura, Takahito Miyake, Hideshi Okada, Hideaki Oiwa, Yosuke Mizuno, Yuichiro Kitagawa, Tetsuya Fukuta, Haruka Okamoto, Masato Shiba, Norihide Kanda, Takahiro Yoshida, Shozo Yoshida, Shinji Ogura

**Affiliations:** Advanced Critical Care Center, Gifu University Hospital, 1-1 Yanagido, Gifu-Shi, Gifu, 501-1194 Japan

**Keywords:** Compartment syndrome, Crush syndrome, Gluteal compartment syndrome, Thigh compartment syndrome, Fasciotomy

## Abstract

**Background:**

Compartment syndrome commonly occurs in patients with forearm and lower leg fractures. Compartment syndromes of the gluteal and thigh muscles are less common. It is imperative that compartment syndrome be diagnosed and treated with fasciotomy as soon as possible. However, there are few reports on the diagnosis and treatment strategies for compartment syndromes that occur simultaneously in multiple anatomical regions or in the ipsilateral gluteal region and thigh.

**Case presentation:**

We report on a 76-year-old man who was obliquely crushed under a tree extending from the right forearm to the left groin. He was brought to our emergency room, where he was diagnosed with compartment syndrome of the right forearm and left lower leg and crush syndrome. Emergency fasciotomy was performed. On the day after admission, swelling and tightness of the left gluteal thigh became apparent, and intracompartmental pressures were elevated, which led to an additional diagnosis of these compartment syndromes. A fasciotomy was performed, the gluteal skin incision was made according to the Kocher–Langenbeck approach (one of the posterior approaches for hip fractures), and the thigh was approached by extending the incision laterally. This surgical approach enabled the decompression of the compartments through a single incision and allowed for easier wound treatment and closure.

**Conclusion:**

This case highlights the diagnosis and treatment of compartment syndrome in four anatomical regions. Extension of the Kocher–Langenbeck approach to the lateral thigh can be a useful surgical approach for ipsilateral gluteal and thigh compartment syndrome.

**Supplementary Information:**

The online version contains supplementary material available at 10.1186/s12245-024-00675-5.

## Background

Compartment syndrome is an emergency condition, for which early diagnosis and treatment are crucial. Delayed interventions can lead to various irreversible complications including necrosis of muscles and nerves, crush syndrome, infection, and gait disorder [1, 2]. Traditionally, the “6Ps” which includes pain, pallor, paresthesia, paralysis, poikilothermia, and pulselessness are known, but rarely are they all present [2, 3]. If compartment syndrome cannot be ruled out, the intracompartmental pressure should be measured [4]. Once the diagnosis of compartment syndrome is confirmed, fasciotomy should be performed immediately to decompress the compartment [5].

Compartment syndrome is more common in the lower leg and forearm, while compartment syndrome of the gluteus and thigh is rare [1, 6, 7]. Compartment syndrome is often caused by traumatic fractures (approximately 75%) [4] but can also be caused by vascular injury, prolonged compression, ischemia, or venous thrombosis [8–11]. Several case reports of compartment syndrome occurring simultaneously in multiple anatomical regions have been published [12, 13], but the frequency and preferred site remain unknown.

We encountered a case of compartment syndrome of the forearm and another side of the lower leg, gluteal, and thigh, which is the first case report of compartment syndrome in four anatomical regions. The gluteal and thigh compartments were released from one incision to facilitate subsequent wound care and closure. Herein, we report the results of our treatment.

## Case presentation

**Fig. 1:**
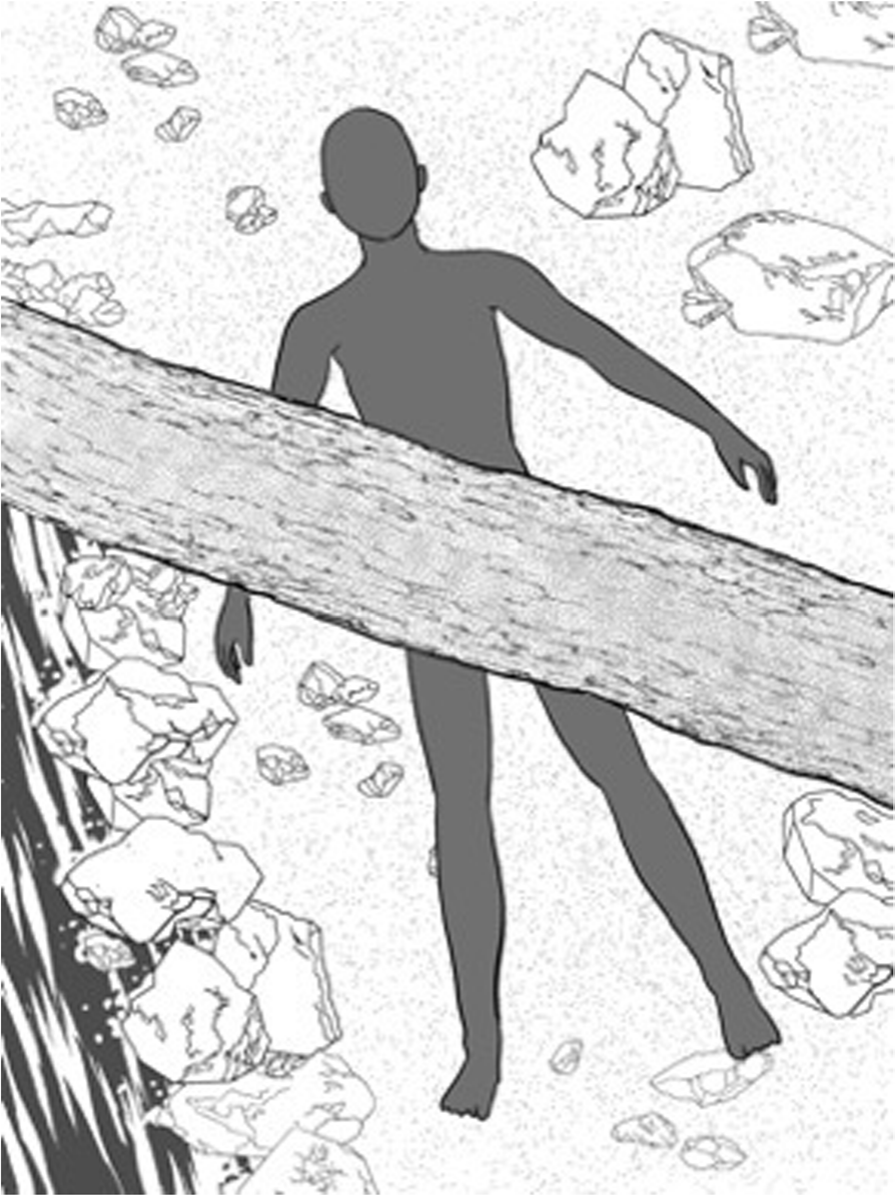
Depiction of the injury mechanism based on details from the patient's interview. He was crushed under the tree obliquely from the right forearm to the left groin in a supine position

A 76-year-old man was fishing in a stream the day after a heavy rain when a tree approximately 40 cm in diameter fell on him. In the supine position, he was pinned under a tree from his right forearm to his left groin and was unable to move (Fig. 1). The patient was rescued 10 h after the accident and transported to the nearest emergency hospital. He was transferred to our emergency room 15 h after the accident for further examination and intensive care. He had no significant medical history and was not taking any medication.

**Fig. 2:**
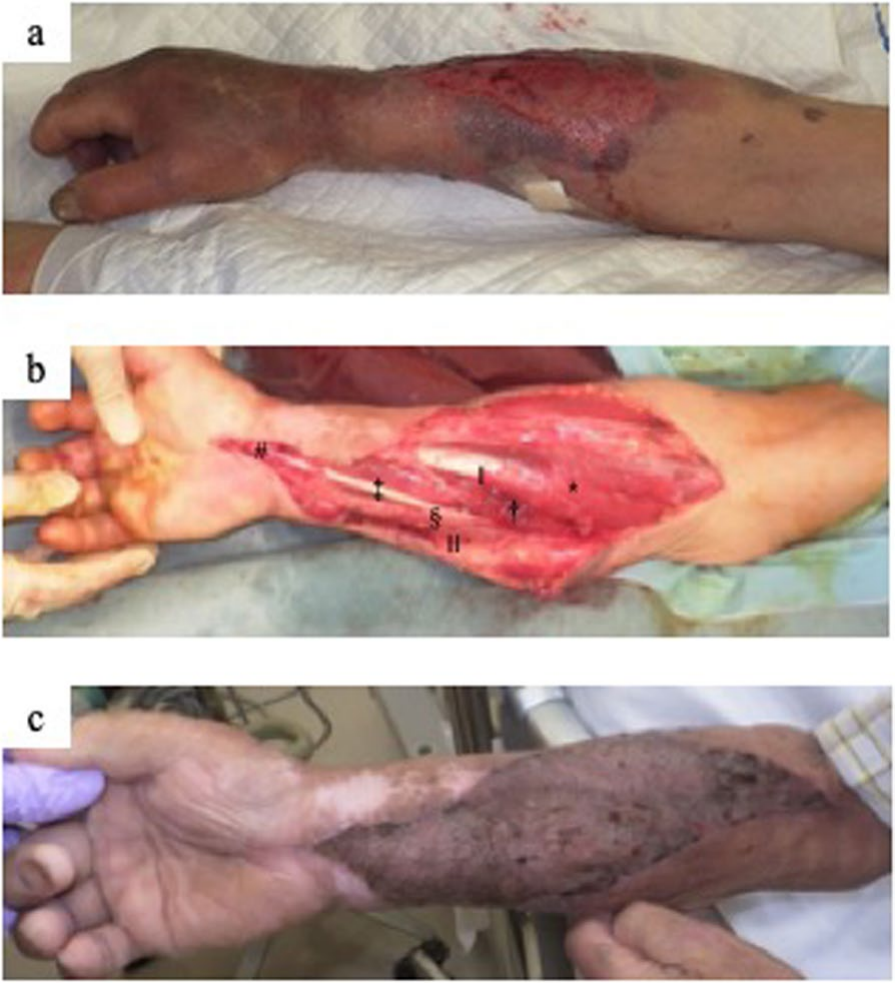
Right forearm compartment syndrome, fasciotomy, and closure. **a** Right forearm 15 h after the accident with painful swelling, purpura, and epidermal exfoliation. **b** One-incision fasciotomy. The flexor digitorum superficialis revealed poor coloration. **c** Wound closure with split-thickness skin grafts (STSGs). *: flexor carpi radialis, †: flexor digitorum superficialis, ‡: palmaris longus, §: flexor carpi ulnaris, #: carpal tunnel, ‖: volar compartment, ‖‖: mobile compartment

**Fig. 3:**
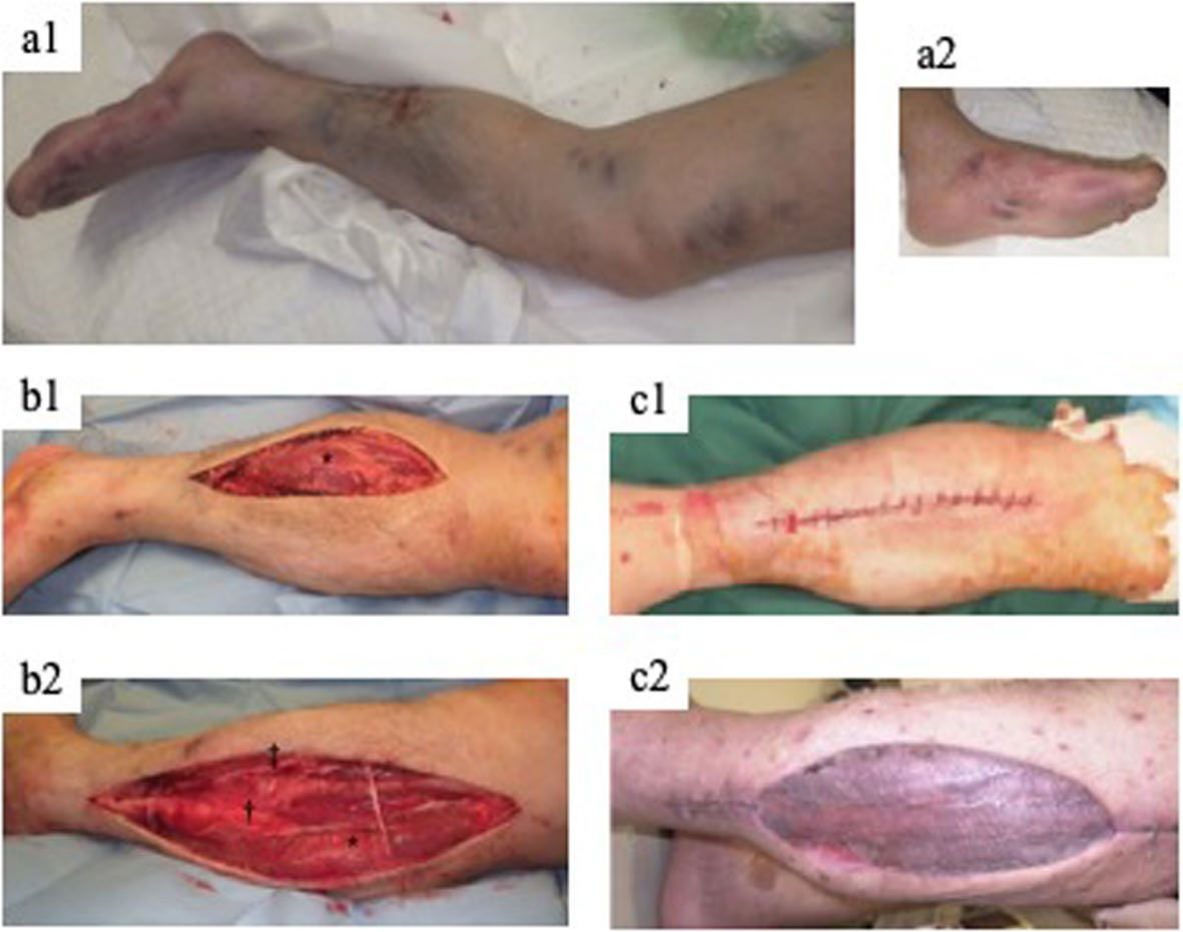
Left lower leg compartment syndrome, fasciotomy, and closure. a1 and a2 Left lower leg and left foot 15 h after the accident, respectively, with painful swelling and purpura on the lower legs and purpura and blistering on the foot. b1 and b2 Two-incision fasciotomy; b1 medial incision and b2 lateral incision. c1 and c2 Wound closure; c1 delayed primary closure on the medial incision and c2 closure with split-thickness skin grafts (STSGs) on the lateral incision. *: superficial posterior compartment, †: lateral compartment, ‡: anterior compartment

On arrival, his vital signs were as follows: respiratory rate, 30 breaths/min; SpO_2_, 98% under 3 L O_2_/min; pulse rate, 94 beats/min; blood pressure, 114/82 mmHg; Glasgow coma scale, 13 (eye, 3; verbal, 4; motor, 6); body temperature, 36.6 centigrade. Physical examination revealed swelling and pain in the right forearm and left lower leg (Figs. 2-a and 3-a1). Purpura and blistering were observed on the left foot (Fig. 3-a2). Blood tests showed a marked elevation in muscle enzymes. Serum potassium and lactate levels were elevated. Table 1 presents the results of the laboratory examinations. He was diagnosed with compartment syndrome of the right forearm and left lower leg based on his symptoms, as well as crush syndrome. Radiographic examination revealed a left diaphyseal ulnar fracture and left distal clavicle fracture, which were treated conservatively. No fractures were observed in the radius, pelvis, or lower extremities.

**Table 1:** Laboratory findings at the time of transfer to our hospital

**Biochemical tests**					
TP	3.4	g/dL	Na	138	mmol/L
Alb	1.8	g/dL	K	5.6	mmol/L
CK	69,613	U/L	Cl	107	mmol/L
AST	525	U/L	Ca	5.2	mg/dL
ALT	160	U/L	P	7.3	mg/dL
LD	1040	U/L	Mg	2.4	mg/dL
ALP	120	U/L	UA	13.2	mg/dL
γGTP	13	U/L	Glu	332	mg/dL
ChE	189	U/L	HbA1c	5.9	%
Amy	923	U/L	T-Bil	0.4	mg/dL
Cre	1.18	mg/dL	CRP	2.48	mg/mL
BUN	24.2	mg/dL	Mb	172,494	ng/mL
**Blood cell counts**					
WBC	12,060	/uL	MCV	96.0	fL
Hb	12.2	g/dL	Plt	176,000	/uL
Ht	12.2	g/dL			
**Coagulation tests**					
APTT	29.4	sec	FDP	332.5	ug/mL
PT	75	%	D-dimer	85.7	
Fib	67	Mg/dL	ATIII	48	%
**Blood gas analysis (3 L/min oxygen mask)**					
pH	7.350		HCO3-	16.9	mmol/L
PaCO2	31.3	mmHg	BE	-7.3	
PaO2	118	mmHg	Lac	64	mg/dL

*TP* total protein, *Alb* albumin, *CK* creatine kinase, *AST* aspartate transaminase, *ALT* alanine transaminase, *LD* lactate dehydrogenase, *ALP* alkaline phosphatase, *γGTP* γ-glutamyltransferase, *ChE* cholinesterase, *Amy* amylase, *Cre* creatinine, *BUN* blood urea nitrogen, *UA* uric acid, *Glu* glucose, *HbA1c* hemoglobin A1c, *T-Bil* total bilirubin, *CRP* C-reactive protein, *Mb* myoglobin, *WBC* white blood cell, *Hb* hemoglobin, *Ht* hematocrit, *MCV* mean corpuscular volume, *Plt* platelet, *APTT* activated partial thromboplastin time, *PT* prothrombin time, *Fib* fibrinogen, *FDP* fibrinogen/fibrin degradation products, *ATIII* antithrombin III, *pH* potential hydrogen, *PaCO2* partial pressure of carbon dioxide, *PaO2* partial pressure of oxygen, *HCO3* bicarbonate ion, *BE*, base excess, *Lac* lactate

The patient was intubated in the emergency room and admitted to an advanced critical care center. After admission, he required immediate blood purification therapy (hemodiafiltration) for crush syndrome. Simultaneously, fasciotomies of the right forearm and left lower leg were performed in the emergency department. On the right forearm, a solitary incision was made on the volar side from the medial side of the elbow joint to the radial side of the wrist joint. The flexor digitorum superficialis muscle revealed poor coloration. The volar and mobile compartments were decompressed, but the dorsal compartment was not released based on the findings of swelling and tension (Fig. 2-b). Two incision-leg fasciotomies (medial and lateral) were performed on the left lower leg. All the compartments (anterior, lateral, deep, and superficial posterior) were decompressed. Swelling in the anterior, lateral, and superficial posterior compartments was particularly intense (Fig. 3-b).

**Fig. 4:**
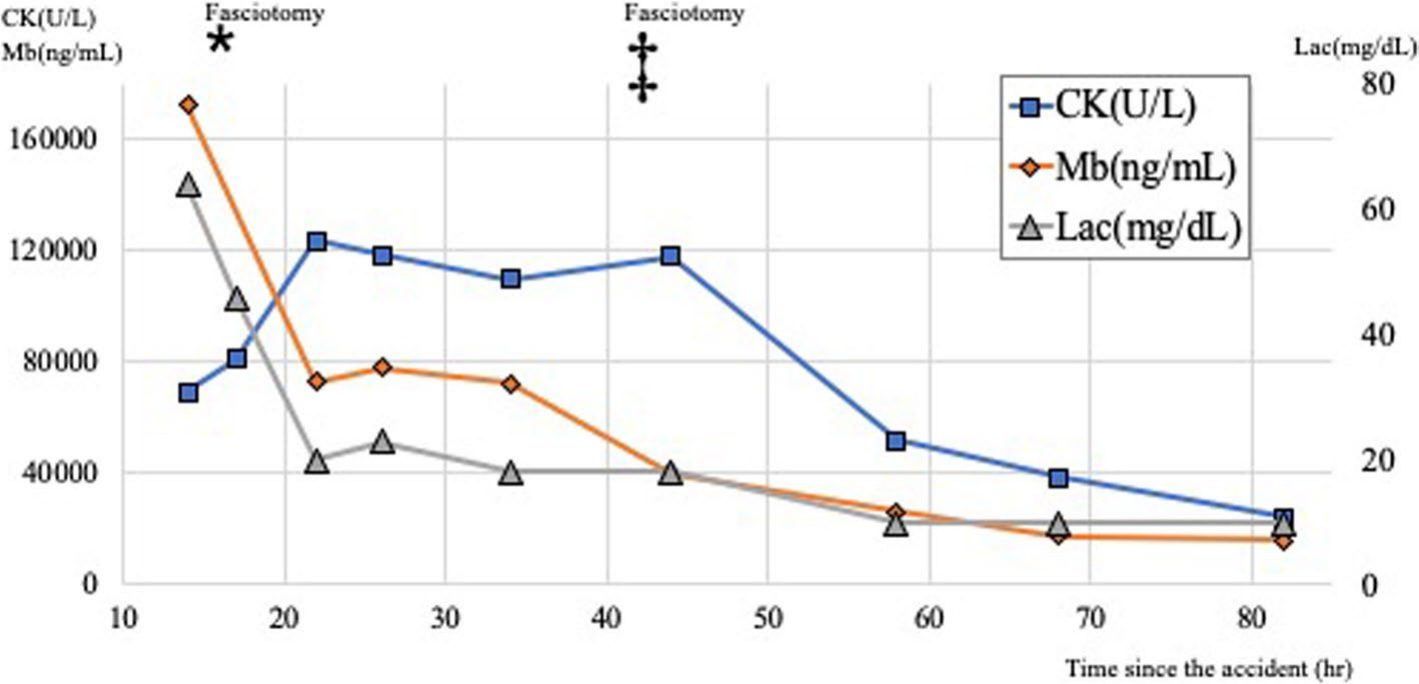
Changes in serum CK, Mb, and Lac levels The left vertical axis of the graph shows CK (U/L) and Mb (ng/mL) values, the right vertical axis shows Lac values, and the horizontal axis shows the time (h) since the accident. CK, creatine kinase; Mb, myoglobin; Lac, lactate; h, hour. ⁎: Fasciotomy of the right forearm and left lower leg compartment syndrome. ‡: Fasciotomy of the left gluteal and left thigh compartment syndrome

**Fig. 5:**
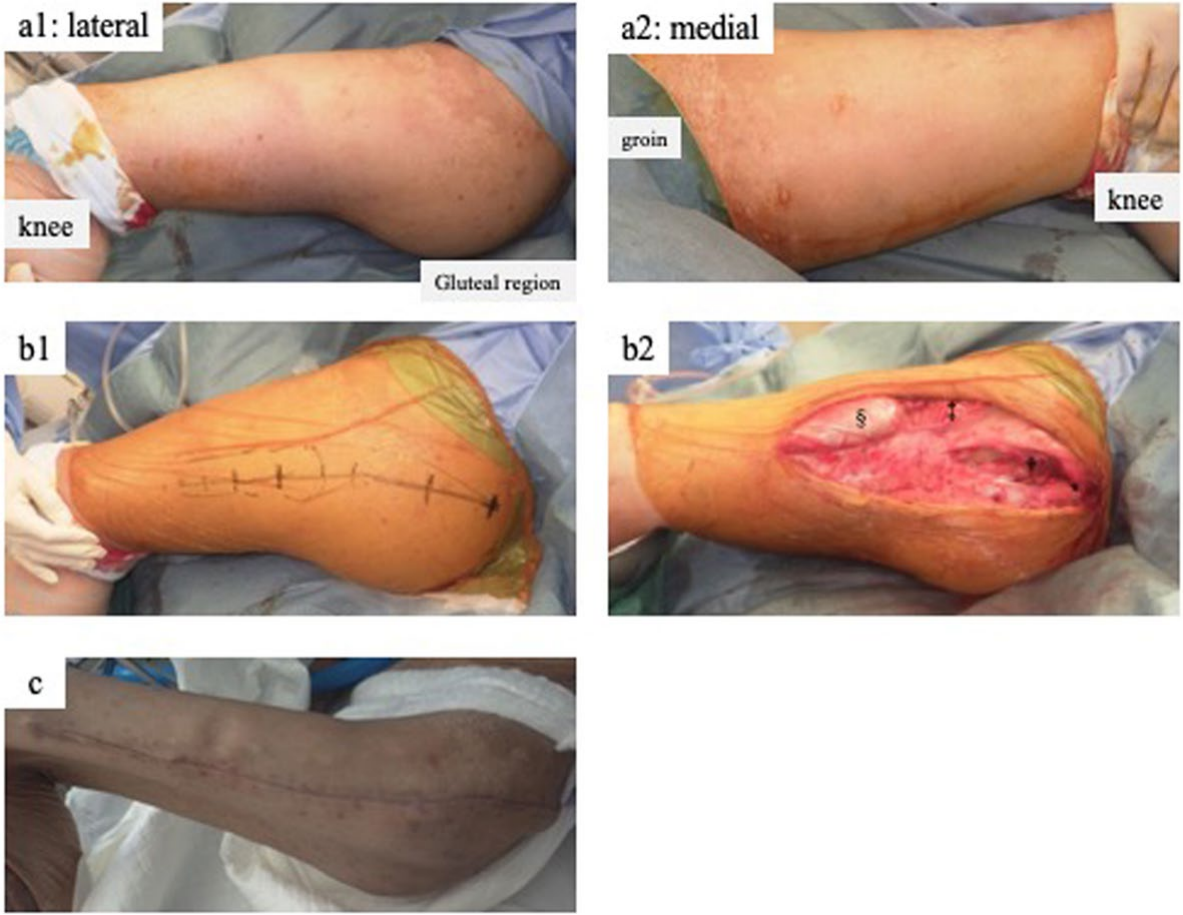
Left gluteal and thigh compartment syndrome, fasciotomy, and closure. a1 and a2 show the gluteal and thigh regions 40 h after the accident; the gluteal to the thigh region on the left were markedly swollen and tense, and blisters were noted on the medial thigh. b1 Markers of the planned skin incision; the gluteal incision was made according to the Kocher–Langenbeck approach, and the incision was extended over the lateral thigh. Three separate anatomic gluteal compartments composed of the gluteus maximus, gluteus medius/minimus, and tensor fascia lata; and the anterior compartment of the thigh were decompressed. The colorations of the gluteus maximus, gluteus medius, and gluteus minimus were poor. The vastus lateralis was protruded (b2). c Delayed primary closure of the gluteal and thigh regions. *: gluteus maximus, †: gluteus medius, ‡: tensor fasciae lata, §: vastus lateralis

Although massive infusion and continuous hemodiafiltration therapy was continued, the serum creatine kinase level remained elevated even after the initial treatment (Fig. 4). Forty hours after the injury, the left gluteal region of the left thigh was markedly swollen and tense (Fig. 5-a). Intramuscular compartment pressures were measured using an arterial line setup as follows: 56 mmHg in the gluteus maximus, 46 mmHg in the tensor fasciae latae, 19 mmHg in the anterior thigh, and 23 mmHg in the posterior thigh (radial artery blood pressure was 110/58 mmHg). He was diagnosed with compartment syndrome of the left gluteus and thigh, and fasciotomy was performed. A gluteal incision was made according to the Kocher–Langenbeck approach, which is a posterior approach for hip fractures. The incision was extended distally over the lateral thigh (Fig. 5-b1). Three separate anatomical gluteal compartments, the gluteus maximus, gluteus medius/minimus, and tensor fasciae lata, were released. The coloration of the gluteus maximus, gluteus medius, and gluteus minimus was poor. In the thigh, the anterior compartment was released from the same skin incision and the vastus lateralis muscle protruded. The other compartments of the thigh were not released because swelling and tension markedly improved after decompression of the anterior compartment. Thigh muscle color was good (Fig. 5-b2). The intramuscular compartment pressures were not measured post-fasciotomy, as intraoperative findings clearly indicated successful decompression and further invasive procedures needed to be avoided.

No muscle necrosis was observed in the left lower leg. On the 17th day, delayed primary closure was performed on the medial incision wound, and split-thickness skin grafts (STSGs) were performed on the lateral incision (Fig. 3-c). The flexor carpi radialis, flexor digitorum superficialis, and flexor carpi ulnaris gradually became partially necrotic. On the 17th day, the muscles were debrided, and negative pressure wound therapy (NPWT) was initiated. On the 21st day, the flexor carpi radialis and palmaris longus muscles became necrotic and were debrided. STSGs was performed on the 28th day (Fig. 2-c).

In the left gluteus and thigh, parts of the gluteus maximus and gluteus medius were necrotic. On the 17th day, NPWT was initiated following debridement. On the 20th day, delayed primary closure was performed on the thigh and NPWT was continued on the gluteal surface. On the 31st day, the remaining wound was successfully closed (Fig. 5-c).

On the 11th day, a tracheostomy was performed. Subsequently, sedative medication was discontinued, and despite delirium, the patient was able to communicate. He was weaned from the ventilator on the 16th day. Due to acute kidney injury, the patient received acute blood purification therapy until the 26th day. Throughout the admission, frequent wound observation and lavage were conducted, with wound cultures revealing the presence of several bacteria (see Additional file 1). Antibiotics were administered for wound infection and bacteremia. The patient's overall condition improved, leading to discharge from the advanced critical care center on the 43rd day. The patient’s treatment course is illustrated in Additional file 2.

After transfer to a rehabilitation hospital, the patient was discharged on the 144th day. After discharge, he was living independently without any support and enjoyed hobbies such as mountain stream fishing and running a vegetable garden. The patient’s photographs at the 3-year follow-up are shown in Additional file 3.

## Discussion

### Compartment syndrome of the gluteal and thigh

Gluteal compartment syndrome is often caused by long-term immobilization, and some have been reported to be caused by surgical positioning [14, 15]. The gluteus comprises three compartments: the gluteus maximus, the gluteus medius/minimus, and the tensor fasciae lata [16]. Surgical decompression is usually performed by the ilium posterior approach [14, 17, 18]. The Kocher–Langenbeck approach is the most commonly performed surgical technique [7]. Thigh compartment syndrome is often caused by blunt trauma, and exercise-induced cases have been reported [19]. The thigh consists of three compartments: the anterior, the posterior, and the medial [10, 20]. Surgical approaches often involve unilateral fasciotomies [9, 19].

In this case, the diagnosis of gluteal and thigh compartment syndromes was confirmed the day after the admission. This may be because the symptoms and physical findings of gluteal and thigh compartment syndromes were relatively minor on arrival because they had larger compartment volumes than the forearm and lower leg [20]. The patient was crushed by a tree crossing diagonally from the right forearm to the left groin; therefore, injuries around the groin should have been carefully assessed.

### Treatment strategy for gluteal and thigh compartment syndrome

To the best of our knowledge, there are few reports on concomitant gluteal and thigh compartment syndromes [12, 13]. McNamee et al. report a case of concomitant gluteal and thigh compartment syndrome following prolonged immobilization secondary to alcohol and intravenous drug intoxication [12]. The intramuscular compartment pressures in the right buttock and lateral right thigh were elevated at 50 mmHg and 48 mmHg, respectively. Similar to our case, they made the incision according to the Kocher–Langenbeck approach (referred to as “an incision from the posterior superior iliac spine down to the greater trochanter of the femur and extended to the lateral femoral condyle”), and there was no evidence of muscle necrosis. In addition, Gavriilidis et al. reported a case of combined gluteal and posterior thigh compartment syndrome following accidental fall without a fracture [13]. They noted that fasciotomy of the gluteal and posterior thigh compartments was performed under spinal anesthesia, but the approach and detailed wound condition were uncertain. Of note, the most appropriate surgical approach for ipsilateral gluteal and thigh compartment syndrome remains unknown.

For decompression of the gluteal and thigh compartment syndrome in this case, the incision of the Kocher–Langenbeck approach was extended to the thigh. This skin incision was sufficient to reach a total of six compartments: three each in the gluteal and thigh muscles. This approach makes wound management relatively straightforward. For example, NPWT can be applied to the gluteus and thigh simultaneously from a single incision site. We believe that this one-incision fasciotomy from the gluteus to the thigh is very effective for treating ipsilateral gluteal and thigh compartment syndromes.

In conclusion, we report a case of compartment syndrome in the right forearm, left gluteal region, left thigh, and left lower leg. The one-incision approach is useful for surgically opening the ipsilateral gluteal and thigh compartments.

### Supplementary Information

Additional file 1. Detected microorganisms. The microorganisms detected in the wound and blood are listed, primarily Gram-negative rods.

Additional file 2. Outline of treatment until discharge from the advanced critical care center. Intensive care progresses, such as surgery, ventilatory management, and acute blood purification therapy, are outlined.

Additional file 3. Photographs at 3-year follow up. a, b1, b2, and c show the right forearm, medial left lower leg, lateral left lower leg, and left gluteal–thigh, respectively. d: The patient was able to dress himself.

## Data Availability

No datasets were generated or analysed during the current study.

## Abbreviations

NPWT: Negative pressure wound therapy
STSGs: Split-thickness skin grafts
