# Supplementary material for: Treatment strategy for compartment syndrome at multiple regions due to injuries caused by a tree fall: a case report
Source: Int J Emerg Med. 2024 Jul 15;17:89. doi: 10.1186/s12245-024-00675-5 (PMC11250945; doi:10.1186/s12245-024-00675-5)
Supplement: Supplementary file 1 — Additional file 1. Detected microorganisms. The microorganisms detected in the wound and blood are listed, primarily Gram-negative rods. [file 12245_2024_675_MOESM1_ESM.docx]

Additional file 1. Detected microorganisms

| Date | Sites | Microorganisms |
| --- | --- | --- |
| Day 4 | Right Forearm | *Enterobacter cloacae complex*, *Acinetobacter baumannii*, *Aeromonas hydrophilia*, *Acinetobacter species*, *Stenotrophomonas maltophilia* |
|  | Left Gluteus | *Acinetobacter baumannii*, *Enterobacter cloacae complex* |
|  | Left Lower Leg | *Acinetobacter baumannii*, *Bacillus cereus group* |
| Day 8 | Right Forearm | *Acinetobacter species*, *Stenotrophomonas maltophilia* |
|  | Left Thigh | *Acinetobacter species*, *Stenotrophomonas maltophilia* |
| Day 10 | Blood | *Acinetobacter baumannii* |
|  | Right Forearm | *Acinetobacter species*, *Stenotrophomonas maltophilia* |
|  | Left Thigh | *Acinetobacter species*, *Stenotrophomonas maltophilia* |
| Day 13 | Blood | *negative* |
| Day 14 | Right Forearm | *Staphylococcus haemolyticus (MRCNS)* |
| Day 20 | Blood | *Acinetobacter baumannii* |
| Day 24 | Blood | *negative* |
|  | Right Forearm | *Stenotrophomonas maltophilia* |
|  | Left Gluteus | *Stenotrophomonas maltophilia*, *Candida albicans* |
|  | Left Thigh | *Stenotrophomonas maltophilia* |

MRCNS, methicillin-resistant coagulase-negative Staphylococci
