# Supplementary material for: Treatment strategy for compartment syndrome at multiple regions due to injuries caused by a tree fall: a case report
Source: Int J Emerg Med. 2024 Jul 15;17:89. doi: 10.1186/s12245-024-00675-5 (PMC11250945; doi:10.1186/s12245-024-00675-5)
Supplement: Supplementary file 2 — Additional file 2. Outline of treatment until discharge from the advanced critical care center. Intensive care progresses, such as surgery, ventilatory management, and acute blood purification therapy, are outlined. [file 12245_2024_675_MOESM2_ESM.docx]

Additional file 2. Outline of treatment until discharge from the advanced critical care center

| Date | Event and Treatment |
| --- | --- |
| Day 0 | Suffered the injury |
| Day 1 | Rescued and transferred  Diagnosed with compartment syndrome of the right forearm and left lower leg, crush syndrome, acute kidney injury, and rhabdomyolysis  Intubated  Admitted to the advanced critical care center  Initiated acute blood purification therapy (HDF and CHDF)  Underwent fasciotomies of the right forearm and left lower leg |
| Day 2 | Diagnosed with compartment syndrome of the left glutes and thigh  Underwent fasciotomies of the left glutes and thigh |
| Day 4 | Terminated CHDF and replaced with daily HDF |
| Day 5 | Initiated NPWT on the left lower leg |
| Day 9 | Transitioned to HDF every other day |
| Day 11 | Underwent tracheotomy |
| Day 16 | Weaned from the ventilator |
| Day 17 | Underwent delayed primary closure on the medial left lower leg and STSGs on the lateral left lower leg  Debrided parts of the flexor carpi radialis, flexor digitorum superficialis, flexor carpi ulnaris, gluteus maximus, and gluteus medius  Initiated NPWT on the right forearm and left gluteal thigh |
| Day 20 | Underwent delayed primary closure on the left thigh |
| Day 21 | Debrided the flexor carpi radialis and palmaris longus muscles |
| Day 26 | Terminated acute blood purification therapy |
| Day 28 | Underwent STSGs on the right forearm |
| Day 31 | Underwent delayed primary closure on the left gluteus |
| Day 43 | Discharged from the advanced critical care center |

HDF, hemodiafiltration; CHDF, continuous hemodiafiltration; NPWT, negative pressure wound therapy; STSGs, split-thickness skin grafts
