# Supplementary figures and images for: Treatment strategy for compartment syndrome at multiple regions due to injuries caused by a tree fall: a case report
Source: Int J Emerg Med. 2024 Jul 15;17:89. doi: 10.1186/s12245-024-00675-5 (PMC11250945; doi:10.1186/s12245-024-00675-5)

## Slide 1
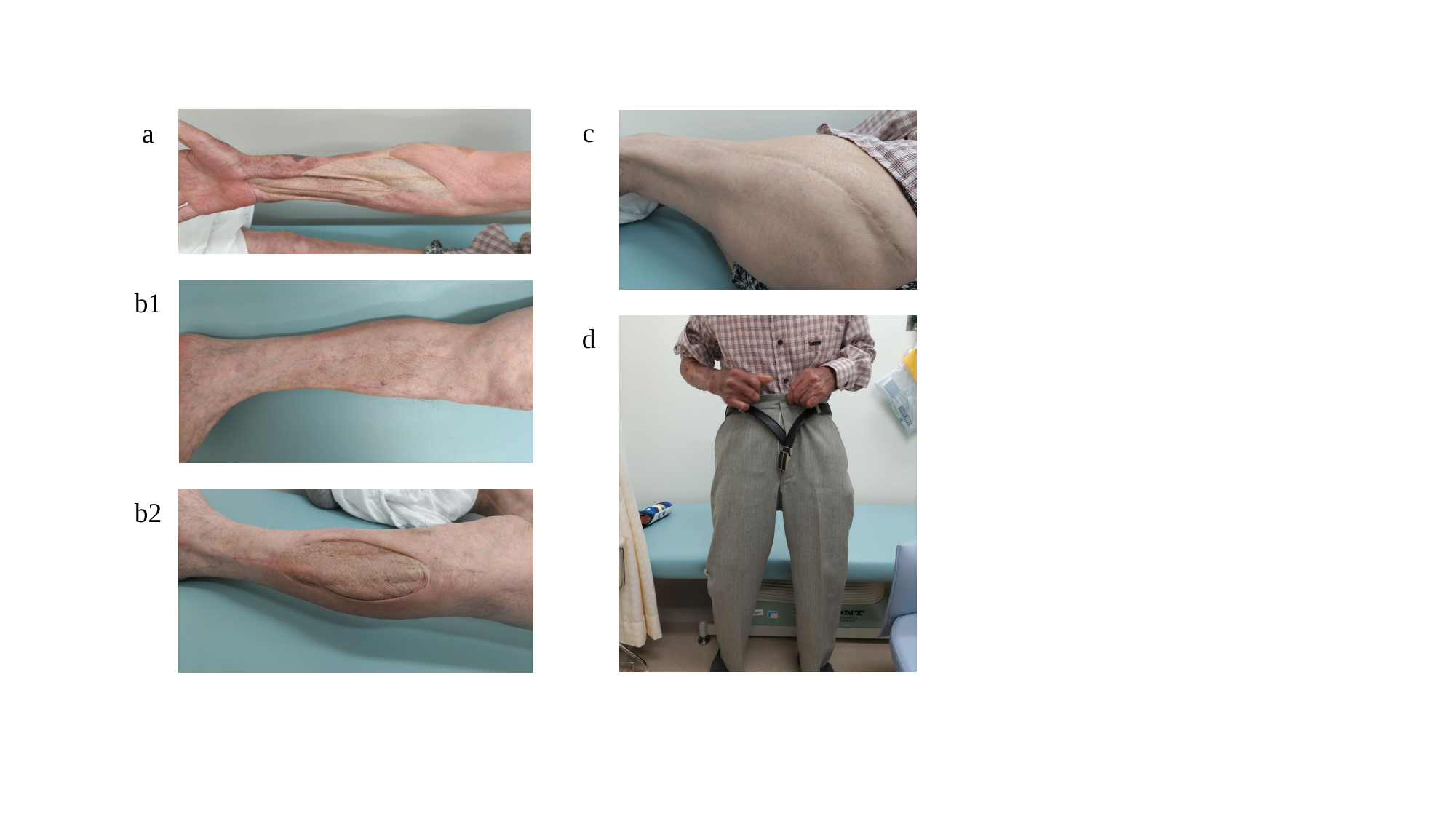

c
a
b1
d
b2

Supplement: Supplementary file 3 — Additional file 3. Photographs at 3-year follow up. a, b1, b2, and c show the right forearm, medial left lower leg, lateral left lower leg, and left gluteal–thigh, respectively. d: The patient was able to dress himself. [file 12245_2024_675_MOESM3_ESM.pptx]
